# The cross-talk between enterocytes and intraepithelial lymphocytes

**DOI:** 10.1186/s40348-016-0048-4

**Published:** 2016-06-01

**Authors:** Serena Vitale, Stefania Picascia, Carmen Gianfrani

**Affiliations:** Institute of Protein Biochemistry, CNR, Via Pietro Castellino, 111, 80131 Naples, Italy

**Keywords:** Intestinal intraepithelial lymphocytes, Enterocytes, Celiac disease

## Abstract

The gut mucosa is continuously exposed to food and microbial antigens. Both enterocytes and intraepithelial lymphocytes have a pivotal role in maintaining the integrity of intestinal mucosa, as these cells guarantee a first line of defense against pathogens and toxic molecules. Enterocytes maintain a physical barrier against microbes and directly contribute to the gut homeostasis by sampling the luminal agents through several pattern recognition receptors or presenting antigen to mucosa T cells. Similarly, due to a close physical contact with the intestinal epithelial cells, the intraepithelial lymphocytes represent an important part of the gut lymphoid tissue, contrasting the entry and spread of pathogens. An alteration of the cross-talk between intestinal epithelial cells and intraepithelial lymphocytes might actively contribute to the development of intestinal immune disorders, as occurring in patients with celiac disease. In genetically predisposed individuals, the gluten exposure results in a massive production of interleukin-15, activation of intraepithelial lymphocytes, and modification of small intestinal mucosa architecture and function. We will review the recent studies on the pathophysiology of cross-talk between enterocytes and intraepithelial T cells, and how this interaction is crucial for intestinal integrity and homeostasis.

## Intestinal lymphoid tissue

The gut-associated lymphoid tissue (GALT) is the largest immune compartment of the body, due to the high cellular number and tissue dimension [1]. The GALT is structured in both organized tissues that comprise Peyer’s patches (PP) and mesenteric lymph nodes (MLNs), and in diffuse lymphoid tissues, with immuno-competent cells scattered in both the epithelium and lamina propria of the throughout intestinal mucosa [2].

The GALT is daily in contact with a large number of harmful and innocuous antigens. In healthy condition, it efficaciously distinguishes between pathogenic antigens, to which an active immune reaction is needed, and non-pathogenic ones, such as commensal bacteria and food antigens, to which an inflammatory reaction would be deleterious. The GALT unresponsiveness toward innocuous antigens, termed oral tolerance, is a fine balanced and strictly regulated process, and its dysregulation is responsible for development of food allergies and chronic inflammatory disorders [2].

The gut tropism of circulating lymphoid cells is determined by selective interactions between adhesion receptors, expressed on the surface of immune cells, and matched ligands on endothelial cells. In the case of T lymphocytes, these cells are selectively driven from peripheral blood to lamina propria or epithelial compartments, depending on the specific homing receptor they express. In particular, lamina propria homing is determined by high levels of integrin α_4_β_7_, interacting with the ligand MadCAM-1 (mucosal vascular cell adhesion molecule-1) on the vascular endothelium, whilst the epithelium commitment is given by the expression of the integrin α_E_(CD103)β_7_ recognized by E-cadherin ligand on the basolateral surface of enterocytes [2].

## Intestinal epithelium infiltrating lymphocytes: phenotype and function

In the epithelium compartment, the GALT is constituted by a heterogeneous population of T lymphocytes (IELs) whose main role is to maintain immune surveillance. Strongly influenced by several luminal factors, as soluble antigens and microbiota, the phenotype and function of IELs markedly change throughout the intestinal tract [3]. Whilst in the colon the IELs population is mostly composed of T cell receptor (TCR) αβ+ CD4+ T cells, in the small intestine it is mainly consisted of CD8+ T cells, carrying the αβ or γδ TCR. In particular, in the small intestine, 70–80 % of IELs are CD8αβ+ cells expressing TCRαβ+, 10–15 % are TCRαβ+ CD4+ T cells, and less than 10 % of cells carrying TCRγδ+ (either CD8+ or double CD8 CD4 negative cells) [3]. The TCRαβ+ CD8αβ+ IELs may express either the inhibitory or co-activator natural killer receptors (C-type lectins NKRs) [3]. In the absence of biological or physical insults, TCRαβ+ IELs have low cytolytic abilities, express inhibitory CD94/NKG2A and low levels of the activating NKG2D receptor, whilst at the same time intestinal epithelial cells do not express ligands for NKRs. Under physiological conditions, the TCRγδ+ IELs have an anti-inflammatory role mediated by the secretion of regulatory cytokines, as IL-10 and TGF-β. In the presence of inflammatory stimuli, the TCRαβ+ IELs up-regulate the activating NKRs, produce pro-inflammatory cytokines, as TNF-α and IFN-γ, and trigger the lysis of infected enterocytes by releasing both perforin and granzyme B and thus preventing the entry and spread of pathogens. Indeed, IELs also participate in the epithelial healing by promoting the intestinal epithelial cell (IEC) growth and epithelial layer repair through the keratinocyte growth factor (KGF) release and by removing infected cells [3].

## Intestinal epithelial cells: emerging role as non-immune inflammatory cells

The IECs form a continuous layer that covers the inside part of the gastrointestinal tract surface. In the past, IECs were considered essentially as non-immune cells with a passive role of physical barrier or target of cytotoxic immune cells. Recent studies have underlined the inflammatory role of IECs, in particular as non-professional antigen-presenting cells. Because of the IECs expressing on their surface the major histocompatibility class (MHC) I and II molecules, as well as non-classical MHC molecules (MIC A/B) whose expression increase in the presence of inflammatory stimuli, they are able to process and to present antigens to both IELs and lamina propria T lymphocytes. Moreover, IECs actively contribute to pathogenic bacteria clearance and to gut homeostasis toward the commensal bacteria, due to the surface expression of pattern recognition receptors (PRRs), as Toll-like receptors (TLRs) and nucleotide-binding oligomerization domains (NODs) [4]. Depending on the intestinal tract sites, the epithelial layer is composed of different types of IECs: enterocytes, goblet, Paneth, and enteroendocrine cells [5]. Each cell subset shows a different morphology and function: the enterocytes have microvilli on their apical surface, whilst the goblet cells contain large vacuoles and secrete mucus; both prevent pathogen invasion. The enteroendocrine cells, scattered throughout the epithelium, produce hormones involved in cellular tropism, tissue repair, and enterocyte differentiation. Interestingly, recent finding suggested that this cell population has a direct role in the gut response to pathogen and commensal bacteria, acting as innate sensor [6]. Finally, the Paneth cells, mainly placed in the crypts, release defensin peptides against pathogens. Though stem cells in the crypts guarantee a continuous and rapid renewal of the intestinal epithelia, alterations in defensin and mucus production, due to luminal insults, damage the integrity of mucosa barrier and may contribute to intestinal immune-mediated disorders, as inflammatory bowel disease (IBD) and celiac disease (CD) [7].

## Enterocytes and T cells in celiac disease

### Adaptive immune response to gluten in the small intestinal mucosa of celiac patients

Celiac disease (CD) is a small intestinal disorder provoked by a dysregulated immune response to wheat gluten and related proteins of barley and rye. This multifactorial disease, caused by both genetic and environmental factors, is characterized by a mild to severe enteropathy and a humoral response to the tissue-transglutaminase autoantigen. HLA class II DQ2 and DQ8 genes are the main risk factors for CD [8] and present gluten immunogenic peptides to lamina propria T lymphocytes, as documented by several studies [9]. Following gluten stimulation, a marked proliferation of Th1 and Th0 lymphocytes, and an increased production of inflammatory cytokines, mainly interferon-γ (IFN-γ), characterize the celiac intestinal mucosa. Up to now, several peptides, derived from gliadins (α-, ω-, and γ-gliadins) and from glutenins, have been identified to stimulate CD4+ T cells in CD subjects [9, 10]. Remarkably, gluten-specific CD4+ T cells can be isolated from the small intestinal biopsies of CD patients and not from non-celiac controls, thus underlining their pathogenic relevance [11].

Despite the pivotal role played by CD4+ T lymphocytes in the pathogenesis of CD, recent evidences suggested the involvement of CD8+ T cells in the inflammatory cascade elicited by gluten. The extensive infiltration of CD8+ T lymphocytes in the mucosa of CD patients, both in the epithelium and lamina propria, represents a histopathological hallmark [12]. Notably, gliadin contains peptides able to bind HLA class I molecules on the surface of antigen-presenting cells [11]. We have found that a short peptide of 10 amino acid length (QLIPCMDVVL), mapping the 123-132 region of α-gliadin stimulates CD8+ T lymphocytes in both peripheral blood and intestinal mucosa of HLA A2-positive celiac patients [13]. A further study with organ culture of celiac mucosal explants has demonstrated that this peptide specifically stimulates cytotoxic CD8+ T cells resident in the lamina propria and induces the apoptosis of enterocytes [14]. Interestingly, initial findings associated the HLA class I A*01 and B*08 genes with susceptibility to celiac disease (CD), though further analysis showed a primary association with alleles encoding for HLA DQ2/8 molecules [15]. By using bioinformatic algorithms for the prediction of peptides with binding motives to surface molecules encoded by A*01 and B*08 genes, we found several sequences in all three main gliadin families that bound to either HLA A1 or B8 molecules with high affinity. Selected peptides also recalled IFN-γ responses in A*01+ and B*08+ CD patients (Picascia et al. manuscript in preparation). A recent genome-wide association study (GWAS) has highlighted the role of B*08 gene, in strong linkage disequilibrium with DR3-DQ2.5, as additional susceptibility factor for CD [16]. Altogether, these findings sustain our previous data on HLA class I-restricted T cell responses in CD and strongly encourage further studies to dissect this, still unexplored, pathway in CD pathogenesis.

### Innate immune response to gluten in the small intestinal mucosa of celiac patients and the role of IL-15

A consistent number of studies have demonstrated that gliadin contains peptides, particular mapping the NH2-terminal part of α-gliadin, able to stimulate innate immuno-competent cells, or even to exert a direct cellular toxicity [17]. After a brief incubation with gliadin peptide p31-43, Maiuri and co-workers reported that the uninflamed CD mucosa up-regulated the expression of ICAM-1, CD69, and HLA-DR markers and the density of interleukin-15 (IL-15)-positive cells. Upon a longer stimulation, an increased expression of CD83 on dendritic cells and of CD25 on T cells and macrophages was observed. This peptide also induced intraepithelial migration of CD8+ T cells in treated CD mucosa and an epithelial damage mainly due to enterocyte apoptosis. Moreover, the same authors found that an early activation of the innate cells might favor the capacity of gliadin peptides, particularly p31-43, to stimulate the adaptive T cell response to immunodominant gliadin epitopes [18]. IL-15 seems to have a key role as mediator of the gliadin-elicited innate activation, as the use of specific neutralizing antibodies inhibited a great part of the above pathways [18].

IL-15 is a pleiotropic pro-inflammatory cytokine, member of the IL-2 family, involved in several mechanisms of both immune and non-immune systems. It is expressed on the surface of mainly monocytes, macrophages, dendritic and epithelial cells in response to inflammatory stimuli. IL-15 has numerous activities, including anti-apoptotic function, stimulation of T cell proliferation, generation of cytotoxic T lymphocytes, and regulation of NK cell survival and function, as well as the induction of immunoglobulin synthesis by B cells. IL-15 is also an important growth and activator factor for both IELs and IECs. In small intestinal biopsies of healthy subjects, IL-15 protein is almost absent in the lamina propria but expressed, though at low intensity, on villous enterocytes. By contrast, in inflammatory condition, as in active CD mucosa, IL-15 is highly expressed by lamina propria mononuclear cells and by intestinal epithelial cells. The overexpression of IL-15 in the epithelium layer has a key role in TCR-independent expansion and activation of IELs in CD mucosa with villous atrophy [19]. Mention et al. reported that IL-15 was massively expressed at the surface of IECs and it was associated with an enhanced transcription of IFN-γ in untreated CD and in refractory celiac sprue patients (RCS), a pathological condition, characterized by massive intraepithelial infiltration with abnormal phenotype and unresponsive to the gluten-free diet [20]. The increased IL-15 levels on enterocytes selectively promoted the growth and activation of clonal CD103+ CD3− IELs in RCS.

Furthermore, it was demonstrated that in CD, IL-15 induced on the epithelial cells similar effects to those elicited by gliadin, as the TFR and FAS expression in both crypt and villous enterocytes, and the up-regulation of Ki67 expression, a marker of cell proliferation. Overall, these results suggested that gluten-induced IL-15 has a pivotal role in the epithelial layer and lamina propria modifications in CD mucosa [21].

There is a general consensus that the mechanisms involved in the villous atrophy in patients with full-blown CD include both the intensive epithelium infiltration by T cells and the increased expression of IL-15, and of non-classical MHC class I molecules, as MICA and MICB, on IECs. MICA and MICB proteins are ligands for the activating NKG2D receptors expressed on the surface of both TCRαβ and TCRγδ CD8+ T cells. MICs act as cellular stress signals and their recognition by NKG2D receptors induces several immune mechanisms, such as cellular cytotoxicity, proliferation, and cytokine secretion. IECs in the inflamed intestinal mucosa of CD patients show an increased expression of non-classical MHC class I molecules. At the same time, the IELs from CD subjects express high levels of the activating NKG2D and CD94/NKG2C receptors and mediate the killing of enterocytes via NKG2D. Additionally, IL-15, by up-regulating the NKG2D receptors, switches on the cytotoxic activity of IEL directed on the neighbor IECs [22]. As previously mentioned, in normal conditions, the cross-talk between IECs and IELs mediated by NKG2D/MIC might maintain the epithelium in a healthy condition eliminating infected cells. By contrast, in the case of uncontrolled IL-15 production by IECs induced by gluten, as in celiac intestinal mucosa, intraepithelial CD8+ T cells are converted into activated killer cells and contribute to epithelial cell destruction and tissue atrophy through a TCR-independent NKG2D signaling pathway [23]. A recent study by Setty and co-workers showed that IECs from healthy subjects with a family history of CD expressed higher levels of IL-15 and heat shock proteins (HSPs) than controls and have ultrastructural epithelial alterations [24]. In addition, they found that the cytotoxic IEL from relatives of CD patients expressed higher levels of activating NK receptors than cells from controls, although at lower levels than patients with active CD. By contrast, IEL from subjects with potential CD failed to up-regulate activating NK receptors, thus suggesting that the fine balance between inhibitory and activating molecules expressed by NK cells is variable in the different forms of the CD. These results re-mark that the innate epithelial stress is an important event to convert IELs into active killer cells and to induce villous atrophy.

## Conclusions

It is becoming clearer that epithelial layer is not only a passive physical barrier against luminal microorganisms and toxic molecules but has a more active role in maintaining the intestinal mucosa integrity. Through a number of surface membrane receptors, enterocytes may act as biosensor of microorganisms preventing their mucosal entry. Furthermore, intestinal epithelial cells take mucosal antigens up and act as non-professional antigen-presenting cells, directly activating the T cell resident both in the epithelium and lamina propria below. Besides the properties mentioned above, the prominent role of enterocytes as non-immune inflammatory cells is emerging in immune-mediated disorders. To throughout dissect the cross-talk between intestinal epithelial cells and lymphocytes of both epithelium and lamina propria compartments, it is needful to understand the physiology of intestinal mucosa, as well as the development of intestinal inflammatory cascade occurring in common, and worldwide growing, intestinal disorders, as CD and IBD.

## Abbreviations

CD, celiac disease; GALT, gut-associated lymphoid tissue; GWAS, genome-wide association study; IBD, inflammatory bowel disease; IECs, intestinal epithelial cells; IELs, intraepithelial lymphocytes; IL-15, interleukin-15; KGF, keratinocyte growth factor; MadCAM-1, mucosal vascular cell adhesion molecule-1; MHC, major histocompatibility class; MLNs, mesenteric lymph nodes; NKRs, natural killer receptors; NODs, nucleotide-binding oligomerization domains; PRRs, patter recognition receptors; RCS, refractory celiac sprue patients; TLRs Toll-like receptors
